# Advances in Risk Management and Screening for Women at Increased Risk of Breast Cancer: The Role of MR Imaging and Personalised Approaches

**DOI:** 10.2463/mrms.rev.2025-0056

**Published:** 2025-10-11

**Authors:** Akane Ohashi, Daniel Förnvik, Ylva Bengtsson, Sophia Zackrisson, Masako Kataoka

**Affiliations:** 1 Department of Translational Medicine, Diagnostic Radiology, Lund University, Malmö, Sweden; 2 Department of Diagnostic Imaging and Physiology, Skåne University Hospital, Malmö, Sweden; 3 Department of Diagnostic Imaging and Nuclear Medicine, Kyoto University Graduate School of Medicine, Kyoto, Kyoto, Japan; 4 Department of Translational Medicine, Medical Radiation Physics, Lund University, Malmö, Sweden; 5 Department of Haematology, Oncology and Radiation Physics, Skåne University Hospital, Lund, Sweden; 6 Division of Oncology, Department of Clinical Sciences, Lund University, Lund, Sweden; 7 Preemptive Medicine and Lifestyle-related Disease Research Centre, Kyoto University Hospital, Kyoto, Kyoto, Japan

**Keywords:** high-risk breast cancer screening, magnetic resonance imaging screening, risk management in high-risk breast cancer women

## Abstract

This review focuses on the management of women at high risk for breast cancer, particularly those with pathogenic variants in *BRCA1* and *BRCA2*, who face elevated lifetime risks. We explore preventive strategies such as risk-reducing surgeries and enhanced screening methods, including MRI. MRI has proved to be an effective tool in early cancer detection, particularly in high-risk populations, and represents a shift toward more personalized and precise screening protocols. Considering the rise in awareness and progress in genetic testing, there is a growing demand for tailored screening methods that balance efficacy, accessibility, and sustainability. Technological advances have resulted in the availability of a range of screening options, and this review highlights the potential for continued innovation in clinical practice and the adoption of strategies that take individual risk factors into account to improve long-term outcomes in breast cancer management.

## Introduction

Breast cancer has the highest morbidity among all cancers. In 2022, an estimated 2.3 million new cases (11.6% of all cancers) were reported globally, with 6.9% of these resulting in death, according to population-based cancer registries.^1^ Additionally, breast cancer incidence has been increasing in most countries.^2^ Many countries have established national programs to combat cancer, emphasizing both primary and secondary prevention to reduce cancer-related morbidity and mortality. Primary prevention involves eliminating pathogenic factors, such as lifestyle and environmental elements, associated with the development of breast cancer.^3^ Secondary prevention focuses on preventing further development of malignant tumors through the early detection of cancers. Mammography screening serves as a secondary prevention method, leading to a reduction in breast cancer mortality and improved survival.^4^^–^^6^ According to a review of 14 guidelines, most recommend annual or biannual mammography screening for average-risk women aged 40–74.^7^ However, breast cancer risk varies among women, and those at high risk of breast cancer require dedicated screening strategies to reduce the risk of breast cancer death. This review provides an overview of the preventive management methods for women at high risk of breast cancer with a focus on early cancer detection. As the effective use of image-based screening is crucial for reducing mortality, the contribution of MRI screening to prognosis is discussed. In addition, long-term, continuous screening participation remains a challenge, making it essential to understand and address the factors that affect attendance. We examined how variability in adherence to conventional mammography among average-risk women may inform future strategies to improve adherence to MRI screening.

There is no clear consensus on whether imaging follow-up for at-risk women should be termed “screening” or “surveillance.” While “surveillance” may be more accurate by definition, in this article, we use the term “screening” given the number of uses in published studies. When cited sources use the term ‘surveillance,’ we retain their original wording.

## Breast Cancer Risks

Several statistical models can estimate breast cancer risks using the risk factors including a personal or family history of breast cancer, first-degree relative with breast or ovarian cancer, pathogenic variants (*BRCA1, BRCA2*, etc.), prior chest radiotherapy (ages 10–30), high-risk breast lesions (atypical ductal hyperplasia, atypical lobular hyperplasia, and lobular carcinoma *in situ*), personal hormone and reproductive history, and dense breast tissue.^8^ While most breast cancers occur sporadically, twin studies indicate that 27% of breast cancers have a hereditary component.^9^ The lifetime risk of developing breast cancer is significantly higher among women with inherited risk factors for breast cancer, especially those with the *BRCA1* and *BRCA2* pathogenic variants. The cumulative lifetime risk of breast cancer is estimated at 72% (95% confidence interval [CI], 65%–79%) for *BRCA1* carriers and 69% (95% CI, 61%–77%) for *BRCA2* carriers by the age of 80. An estimated 40% (95% CI, 35%–45%) of *BRCA1* carriers and 26% (95% CI, 20%–33%) of *BRCA2* carriers develop contralateral breast cancer within 20 years following an initial breast cancer diagnosis.^10^ Therefore, it is essential for these women to adopt effective strategies for managing their breast cancer risk. However since risk levels change with age, age and life expectancy must be carefully considered in risk management.^11^

**Fig. 1:**
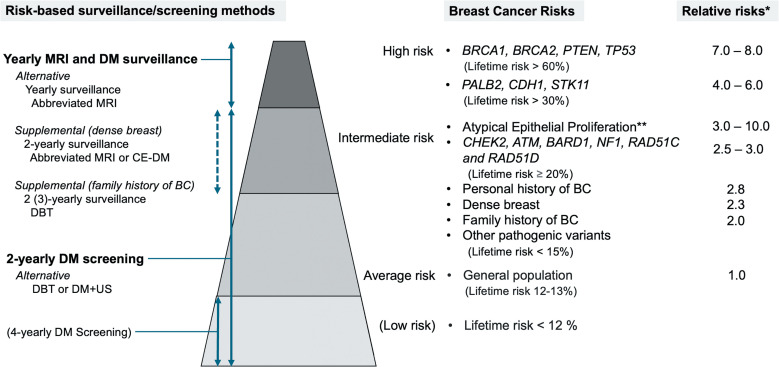
Breast cancer risk stratification and corresponding risk-based screening/surveillance methods, including proposed approaches. Methods shown in bold are currently recommended; those not in bold represent approaches under investigation, which may change in the future. *Relative risks are compared to the general population. **Atypical Epithelial Proliferation includes lobular carcinoma *in situ*, atypical ductal carcinoma, etc. BC, breast cancer; CE-DM, contrast-enhanced digital mammography; DM, digital mammography; DBT, digital breast tomosynthesis; US, ultrasound.

Breast cancer risk levels can be classified into 4 categories based on relative risk (RR) and lifetime risk, as illustrated on the right side of Fig. 1.^12^^,^^13^ According to National Comprehensive Cancer Network (NCCN) guidelines, annual MRI screening is recommended for high-risk women, while those at intermediate risk (pathogenic variants, lifetime risks ≥ 20%) are advised to consider it. For women with other intermediate-risk, there is not enough evidence to recommend MRI screening.^13^ A risk-stratified screening study, My Personal Breast Screening (MyPeBS) trial,^14^ is ongoing. It is a randomized controlled trial across 6 European countries starting from 2019. They use invasive cancer risk at 5 years to divide women into 4 categories and follow-up using risk-based screening methods, including fourth-year mammography screening for lower-risk women (the left side of Fig. 1). The primary outcome measure is evaluating whether a risk-stratified screening approach is non-inferior to standard age-based screening in reducing the incidence of advanced breast cancer (stage 2 or higher). Population-wide risk assessment could be implemented if the risk-stratified screening proves non-inferior to current programs.

## Breast Cancer Risk Reduction Methods in High-risk Women

**Fig. 2:**
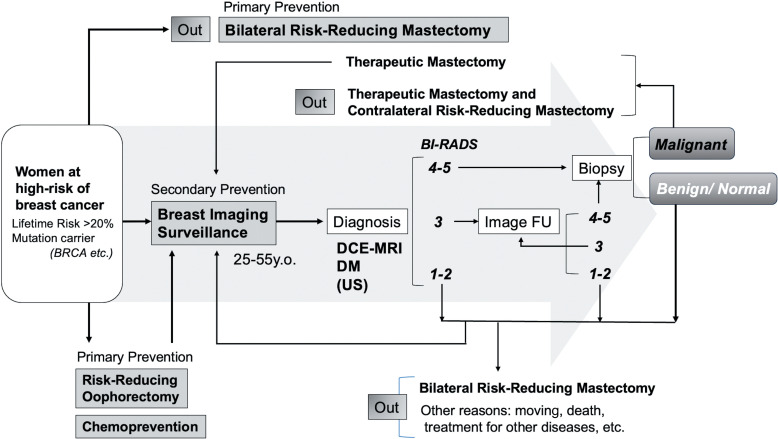
Workflow for women at high risk of breast cancer, illustrating primary and secondary prevention strategies as well as outcomes of breast imaging surveillance.BI-RADS, Breast Imaging Reporting and Data System; DCE-MRI, dynamic contrast-enhanced MRI; DM, digital mammography; FU, follow-up; US, ultrasound.

Understanding the effectiveness of the options to control risk for the carriers of pathogenic variants is vital. Figure 2 illustrates how different choices affect the flow in a hypothetical high-risk screening scenario. Women at increased risk for breast cancer have different strategies to mitigate their risk compared to average-risk women: primary prevention with chemoprevention, risk-reducing mastectomy (RRM), risk-reducing salpingo-oophorectomy (RRSO), and secondary prevention with early cancer detection with MRI and mammography screening. Bilateral and contralateral RRM might be the main reasons for discontinuing screening. In a cohort of high-risk women reported by Haroun et al., the average time from initiation of screening to RRM was 3.6 years.^15^ When women detect cancer during screening, it leads to therapeutic mastectomy and contralateral prophylactic mastectomy. Additionally, we present a diagnostic process based on assessment categories of Breast Imaging Reporting and Data System (BI-RADS, 5th edition, 2013^16^), which reflect the likelihood of cancer based on imaging findings: Category 4–5 recommended to do biopsy confirmation, Category 3 requires 6 months follow-up, and Category 1–2 require continuing routine screening (in this figure, we did not divide Category 4 into the 3 subcategories used in mammography or ultrasound).

A meta-analysis based on 15 unique studies showed that bilateral RRSO and bilateral RRM were associated with decreased breast cancer risk for *BRCA1/2* carriers, with a summary RR of 0.55 (95% CI, 0.45–0.68) and 0.11 (95% CI, 0.04–0.32), respectively. However, bilateral RRM was not associated with a reduction of all-cause mortality rates.^17^ A systematic review showed that RRM prevents breast cancer and decreases mortality in women with *BRCA* mutations (high certainty), but likely has a negative physical impact as complications following surgeries, pain/discomfort, and impact on sexuality (moderate certainty).^18^ Based on the evidence, the NCCN Guidelines Panel advocates discussing RRM with carriers of pathogenic variants individually.^19^ Through patient support and education by healthcare professionals, there is a growing understanding of the importance of long-term risk management. According to a high-risk cohort at an academic center in the USA, carriers of pathogenic variants were 6 times more likely to pursue risk-reducing management (chemoprevention or RRM) compared to those with non-pathogenic variants: hazard ratio (HR) 5.99 (95% CI, 2.63–13.64), *P* < 0.001.^20^ In a recent study from Michigan, USA, 77 women (60%) out of 129 *BRCA1*/*2* carriers underwent RRM.^21^ The decision to undergo RRM should take various factors into account. It will be influenced not only by the knowledge that the woman is a known carrier of pathogenic variants but also by personal preference, family input, and awareness of surgical complications.^22^ Furthermore, the review of the same study suggests that RRM can yield positive effects, such as reduced anxiety, and negative effects, such as impaired body image and perceptions of reduced sexual attractiveness.

A previous study on healthy *BRCA1/2* carriers reported that RRM is associated with a lower breast cancer-specific mortality rate compared to regular imaging-based surveillance, with 1.8 deaths per 1000 person-years observed in the surveillance group and 0.7 in the RRM group, resulting in HR 0.29 (95% CI, 0.02–2.61).^23^ However, since the confidence interval range is wide, it cannot be described as a significant difference. A longer follow-up is warranted to confirm potential differences in survival benefits of the two approaches.

## Imaging Screening for Women with Increased Risk of Breast Cancer

### MRI

Women at high risk of developing breast cancer are more likely to get histologically aggressive high-grade cancers.^24^^,^^25^ In addition, as with cancers in women at all risk levels, the size of the breast cancer at the time of detection may be related to the patient’s prognosis. In a study conducted by van Barele et al. on patients with *BRCA1*/*2* variant-associated breast cancer, smaller tumor sizes were correlated with improved 10 -year overall survival (OS) without chemotherapy, with rates of 90.8% for pT1bN0 and 77.1% for pT1cN0 (*P* = 0.02).^26^ Thus, there is a need for more sensitive technology that can more accurately identify malignant lesions at an earlier stage than conventional mammography-only screening.

Breast MRI became widespread in the middle of the 1990s. After its effectiveness in detecting and diagnosing breast cancers was established, several institutions started prospective, non-randomized screening studies using MRI for high-risk women in the mid to late 1990s. In 2003, the American Cancer Society recommended the use of MRI for screening based on sensitivity data, and guidelines for imaging screening for high-risk women were established.^27^ A study of *BRCA* carriers and women with a lifetime breast cancer risk of 20% or higher found MRI to have a 90.0% sensitivity for detecting breast cancer, compared to 37.5% for both mammography and ultrasound.^28^ A review article assessing the diagnostic performance of breast examination modalities used for high-risk screening found MRI sensitivity ranges from 75.2% to 100%, and specificity ranges from 83% to 98.4%^13^; however, the specificity increased to 90%–97% in subsequent screening rounds.^29^^,^^30^ In addition, several studies have shown that breast cancers identified through screening MRI tend to be smaller and more frequently node-negative (downstaging) compared to those detected by mammography.^30^^–^^32^

### Mammography and ultrasound

Mammography can increase sensitivity by around 5% (0%–8.6%) for high-risk women screened with MRI and decrease specificity by between 0.4% and 2%.^13^ Even a slight decrease in specificity impacts a large number of women in the screening. Therefore, it is still necessary to evaluate how adding mammography to MRI screening can be beneficial and how to maximize its benefits. Sung et al. show that screening with MRI detects more aggressive cancer than mammography screening.^33^ Of cancers detected through MRI screening, 71% (118/167) were invasive, while among those detected by mammography screening, 65% (28/43) were ductal carcinoma *in situ* (DCIS), and 88% (38/43) were identified as calcifications. Nevertheless, the clinical importance of DCIS in the general population remains highly controversial as it may cause overdiagnosis and overtreatment.^34^ A prospective active monitoring trial for DCIS, a non-surgical approach with meticulous imaging to reduce overtreatment, is ongoing. However, key challenges include detecting occult invasive cancer—found in about 25% of DCIS cases at excision^35^—and identifying high-grade DCIS with a high risk of progression to high-grade invasive cancer.^36^

Ultrasound is an effective supplemental tool for mammography screening in women with dense breasts.^37^ On the other hand, no reports indicate that the ultrasound contributes to diagnosis in adjunction to MRI screening. Moreover, adding ultrasound to MRI and mammography screening for high-risk women results in a reduction in specificity of up to 5.5%.^13^ However, when MRI is unavailable, combining the ultrasound with mammography remains a valuable screening strategy.^37^

### Age-specific strategies for MRI and mammography

Hereditary breast cancer tends to occur at a younger age,^38^ and high-risk women diagnosed with breast cancer aged 30 or younger have poor OS.^39^ Thus, the recommended MRI screening starting age in existing guidelines is 25 or 30.^19^^,^^40^ Lowry et al. used a comprehensive simulation model to show that in *BRCA* carriers, an annual MRI from the age of 25 followed by alternating digital mammography (DM)/MRI from the age of 30 might be the most effective screening strategy to provide greater life expectancy and breast cancer mortality reduction.^41^

In a prospective multicenter observational study (EVA trial), the respective contributions of each modality (clinical breast examination, mammography, ultrasound, and MRI) for screening were evaluated using 687 asymptomatic high-risk women (≥20% elevated risk). The percentage of cancers detected by MRI alone (14.9 out of 1000) was the highest. Adding mammography (16.0 out of 1000) did not significantly change the rate, and adding ultrasound did not change the value at all (14.9 out of 1000).^42^ Several reports show that, due to the low contribution of mammography to cancer detection, screening with MRI alone might be effective for high-risk women under 40.^43^^,^^44^ This may be influenced by the high prevalence of dense breasts among women under 40.^43^ The benefits and risks of postponing screening of *BRCA1/2* carriers were also evaluated. According to a meta-analysis of 6 high-risk screening trials, one-third of breast cancers in *BRCA2* carriers under 40 (6/18, 33.3%) could only be detected using mammography alone, including two DCISs. However, in *BRCA1* carriers, mammography has limited added sensitivity for detecting cancers.^45^ A study shows that simulation models estimate that following the current screening protocol prevents 23 (0.17%) more breast cancer deaths per 100,000 *BRCA1* carriers than delaying mammograms until the age of 40; however, delaying mammography until the age of 40 lowers radiation-induced breast cancer deaths by 50%. Considering the radiation risks, the study estimates show the 10-year (30-39 y.o.) screening benefit is minimal or even negative, depending on dose and estimate models.^46^ In addition, this 10-year reduction in mammography screening resulted in significant cost savings of €272900 per life year gained. Further research is needed on optimal strategies of mammographic screening separately for *BRCA1* and *BRCA2*.

### Histology and imaging characteristics of breast cancer in BRCA1 and BRCA2 carriers

**Fig. 3:**
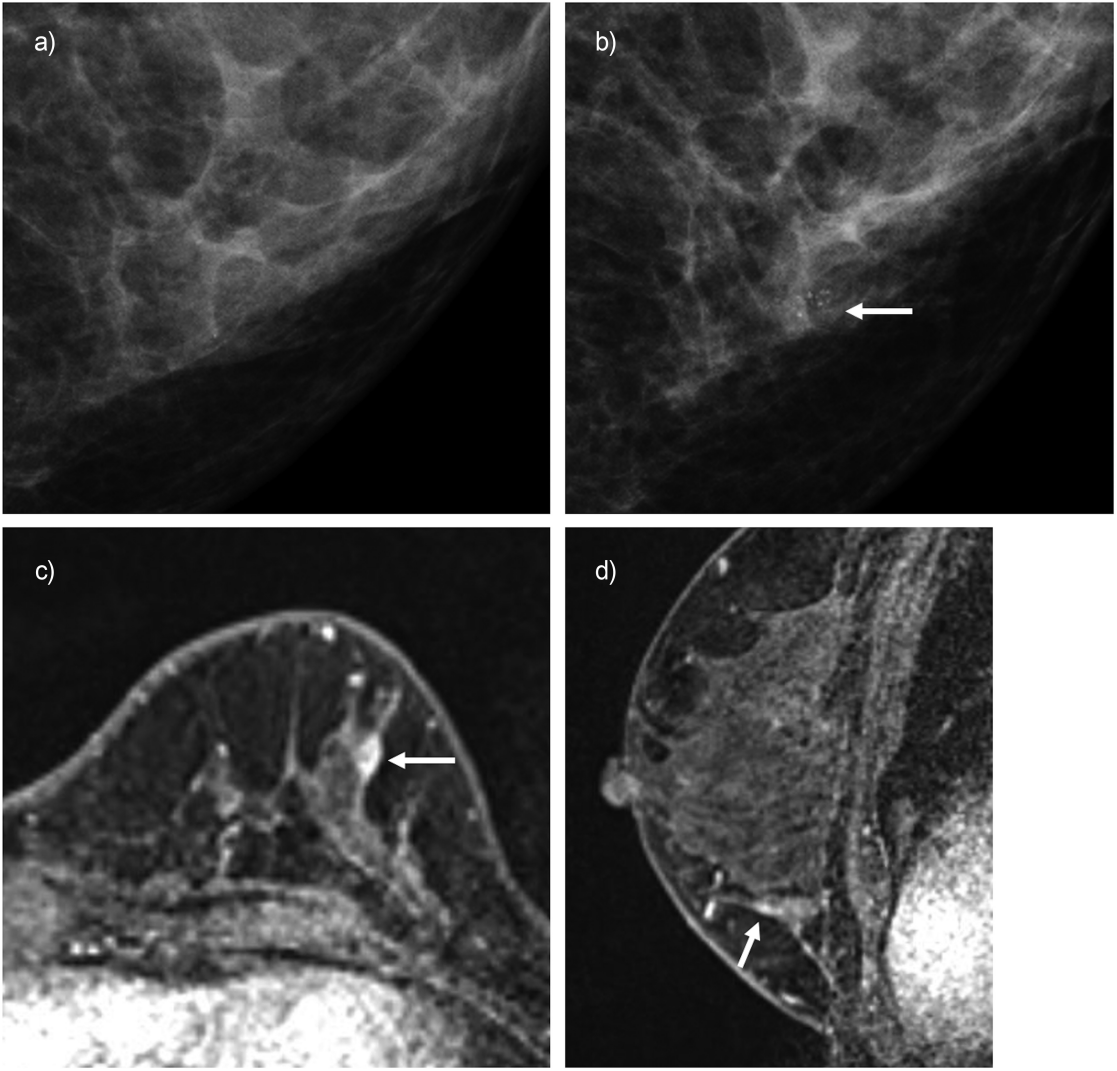
*BRCA2* carrier of pathogenic variants with a right breast-conserving surgery at her 40s. The woman started imaging screening with yearly mammography 14 years after her surgery. (**a**) and (**b**) are the mediolateral oblique mammography images. (**a**) Mammogram was assessed as normal 1 year before the lesion was suspected. (**b**) A clustered calcification appeared in the lower outer quadrant area. (**c**) and (**d**) are the high-resolution DCE-MRI with axial and sagittal images. A 5-mm enhancing lesion was detected and described as BI-RADS Category 4A. The lesion was biopsied and showed DCIS, diagnosed 22 years after she got her first cancer. The white arrows in each image indicate the lesion. BI-RADS, Breast Imaging Reporting and Data System; DCE-MRI: dynamic contrast-enhanced MRI; DCIS, ductal carcinoma *in situ*.

Several studies have provided evidence that the imaging features of MRI and mammography vary by *BRCA* mutation type, reflecting distinct hereditary traits. *BRCA1-*associated cancers exhibited higher nuclear and histological grades compared to *BRCA2-*associated cancers.^32^^,^^47^^–^^50^ Triple negative breast cancer (TNBC) was predominantly observed in *BRCA1* mutation carriers, while *BRCA2* mutation carriers more commonly presented with the luminal phenotype breast cancer or DCIS. *BRCA1*-associated cancers often have MRI findings consistent with the BI-RADS characteristics of TNBC^51^, present as masses with oval shape, well-defined circumscribed margin, and rim-enhancement.^49^^,^^50^ In addition, it tended to develop in the posterior area of the breast.^32^^,^^49^^,^^52^ *BRCA2-*associated cancers often exhibit as calcifications in mammography consistent with DCIS.^32^^,^^47^^,^^52^ *BRCA2*-associated cancers on MRI more commonly present as masses with irregular shapes and spiculated margins and are more likely to exhibit non-mass enhancement (NME) than *BRCA1*-associated cancers.^48^^–^^50^ Figure 3 shows a *BRCA2* carrier woman who underwent imaging screening only using yearly mammography. After 5 screening rounds, a suspicious lesion was suggested at mammography with grouped micro-calcifications. Following MRI assessment of the lesion showing a 5 mm mass, the biopsy revealed a diagnosis of DCIS. Yearly mammography might help in detecting breast cancer for those who are reluctant to undergo MRI screening.

### Imaging screening for dense breast (intermediate risk)

While annual MRI screening is recommended for high-risk women, additional risk populations are being considered for MRI screening. Women with dense breasts, those with a higher proportion of fibroglandular tissue than fatty tissue, exhibit a lower chance of having a cancer detected through mammography because the dense breast tissue may obscure the lesion.^53^ Although women with dense breasts are at lower risk than high-risk women, their risk of developing breast cancer is still about 2.3 times as high as that of normal women,^54^ which puts these women into the intermediate risk group. With MRI, breast lesions can be detected effectively, even in mammographically dense breasts.^55^^,^^56^

A meta-analysis study, including 22 studies, evaluated supplemental modalities for women at average or intermediate risk with dense breasts and negative mammography. They compared handheld ultrasound, automated breast ultrasound, digital breast tomosynthesis (DBT), and MRI and showed that MRI yielded the highest cancer detection rates.^57^ Therefore, this evidence suggests that supplemental MRI screening of women with dense breasts can be considered.

The value of using MRI as a supplemental screening tool is confirmed by a prospective randomized controlled trial, Dense Tissue and Early Breast Neoplasm Screening (DENSE) trial. The study population were women with extremely dense breasts (BI-RADS D) and negative screening mammograms. The trial demonstrated that with biannual MRI the detection rate was 16.5 cancers per 1000 MRI screenings (95% CI, 13.3–20.0) for the first round of the screening.^56^ At the next mammography screening, the cancer detection rate was 2.0 per 1000 in MRI screening participants vs. 7.1 in non-MRI screening participants and 6.0 in the mammography-only group participants, suggesting MRI leading to earlier diagnosis. Furthermore, there was a reduction in interval cancers by 84% (5.0 to 0.8 per 1000 women) in MRI participants who accurately underwent MRI screening compared to the control group with standard screening with biannual mammography. According to the DENSE trial, quadrennial MRI screening is cost-effective, especially among women with extremely dense breast tissue, with a cost of €15620 per quality-adjusted life year (QALY) gained.^58^ While the population differed from high-risk women, this large-scale MRI study in those with dense breasts may help broaden future MRI screening options for high-risk groups.

## Potential Optimization of High-risk Screening Strategies of MRI

**Fig. 4:**
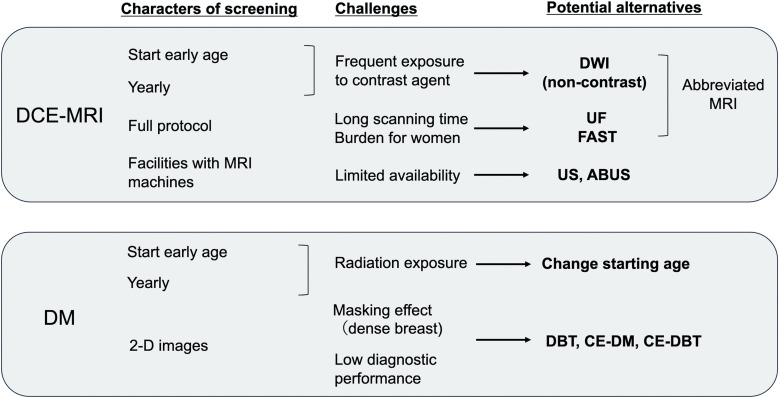
Challenges of standard high-risk screening modalities (DCE-MRI and DM) and proposal of potential alternative modalities. ABUS, automated whole breast ultrasound; CE-DBT, contrast-enhanced digital breast tomosynthesis; CE-DM, contrast-enhanced digital mammography; DBT, digital breast tomosynthesis; DCE-MRI, dynamic contrast-enhanced MRI; DM, digital mammography; DWI, diffusion-weighted image; FAST, first postcontrast subtracted; UF, ultrafast; US, ultrasound.

With the increase in the number of breast and ovarian cancer patients, it is likely that more women, including family members, will be found to have pathogenic variants related to these cancers in the future. This increase is driven not only by a growing number of cancer cases but also by the expansion of genetic testing, including mainstream screening initiatives and broader panel testing, which enhance our ability to detect hereditary risk factors earlier and in a larger population. In addition, if the women carrying pathogenic variants do not decide to undergo RRM, current guidelines recommend annual imaging-based screening starting from the age of 25 or 30.^7^ The number of examinations in this population could be an issue that needs to be considered to reduce future healthcare expenditure. Figure 4 illustrates the relationship between the topics discussed in the recent literature, existing challenges with the nature of screening, and potential alternatives to resolve them. The left side of Fig. 1 illustrates the future risk-based screening methods that can be predicted from recent publications described in this section.

### The potential alternatives to full MRI protocols

An abbreviated MRI protocol with a shorter scanning time could be worth considering for future screening as a cheaper and quicker option, also imposing a reduced burden on women. It was introduced by Kuhl et al. to reduce MRI acquisition time to 3 min and reduce reading time^59^: the protocol consisted of a non-contrast T1-weighted image, a first contrast T1-weighted image, and subtraction and maximum intensity projection images created from these 2 images (first postcontrast subtracted [FAST] protocol). The acquisition time is relatively short compared to the 8–10 min acquisition time of conventional dynamic contrast-enhanced (DCE) MRI. This advanced protocol results in equivalent diagnostic performance to the full diagnostic protocol at a specificity (94.3% and 93.9%, *P* = 0.563, respectively), with 100% sensitivity. A meta-analysis with 5 screening and 8 cohort studies demonstrated that the diagnostic performance of abbreviated MRI protocols is comparable to conventional MRI protocols,^60^ and several studies have evaluated the cost-effectiveness of the abbreviated MRI protocols.^61^ However, abbreviated MRI methods lack functional insights, such as kinetic data from contrast enhancement, which reflects lesion vascularity, and diffusion-based molecular information from DWI, which relates to water mobility and may indicate cellular density, membrane integrity, and tumor aggressiveness. There might be potential to improve performance by adding this multiparametric information.

The ultrafast (UF) protocol is a short scanning protocol introduced by Mann et al. in 2014^62^ that can integrate kinetic information into the abbreviated MRI. Accelerated scanning techniques allow for the acquisition of kinetic information with temporal resolutions of just a few seconds shortly after contrast injection while maintaining spatial resolution; the total scanning time is approximately 1 minute.^63^ This novel technique can extract several kinetic parameters that demonstrate better or comparable diagnostic performance than conventional MRI.^62^^,^^64^^,^^65^ A recent meta-analysis including 16 studies reported on the diagnostic performance of the UF protocol, showing it could differentiate benign from malignant lesions with a sensitivity of 83%, a specificity of 77%, and an area under the receiver operating characteristic curve (AUC) of 0.88.^66^ Furthermore, a study disclosed that morphological information from the UF protocol may have the potential to be used equivalently to conventional MRI in diagnosing invasive mass-shaped breast cancers.^67^ In the screening setting, van Zelst et al. showed comparable sensitivity at UF-only protocols compared to a full multiparametric diagnostic MRI protocol with significantly higher specificity (*P* = 0.002) and 22.8% shorter reading time.^68^ An additional benefit of the UF protocol is its usefulness for individuals with high background parenchymal enhancement (BPE), such as premenopausal women. Since BPE continues to increase after injection, distinguishing from enhancement of malignant lesions is difficult, even in the early phase of conventional MRI.^69^

Another functional information technique that can add molecular information is diffusion-weighted image (DWI). This non-contrast enhanced MRI technique quantifies the random motion of water molecules as the apparent diffusion coefficient. Imaging without contrast agents is an essential option for women allergic to it. Furthermore, some gadolinium agents have been reported to accumulate in the brain.^70^ It is becoming an integral part of routine breast MRI examinations worldwide. The potential use for lesion characterization (distinguishing benign from malignant lesions), treatment response, stratification by breast cancer subtype and prognostic factors has been demonstrated.^71^ Several studies have suggested that diffusion MRI complements DCE-MRI with high diagnostic performance, especially by improving the specificity,^72^ and it helps to avoid biopsies for false positive lesions.^73^^,^^74^ Recent advances in technology have improved spatial resolution, and high-resolution DWI can be used to assess morphological information in similar detail as DCE-MRI.^75^^–^^77^ A study of the combined abbreviated protocol with UF and DWI protocols shows a significantly higher diagnostic accuracy (*P* < 0.0001) due to increased specificity compared to the full MRI protocols.^78^

Kuhl et al. defined several different types of abbreviated MRI protocols,^79^ including UF, DWI, and T2-weighted image (T2WI). The diagnostic performance of all published abbreviated MRI protocols was largely equivalent to the respective full MRI protocols. However, they concluded that there was no evidence to indicate which additional protocols were of value. Regarding this issue, a recent study demonstrates the valuable aspects.^80^ They compared the multireader diagnostic accuracy of various abbreviated MRI protocols with a full multiparametric MRI protocol in women selected from the DENSE trial (intermediate-risk women with dense breasts). The shortest protocol used consisted of dual acquisition of high-temporal low-spatial and low-temporal high-spatial DCE covering the period from before contrast injection through 120s post-injection. Two other abbreviated protocols included DWI and combined DWI and T2WI. Even the shortest protocol showed no significant difference compared to the full protocol: sensitivity (84.3%, 85.9%; *P *= 0.68) and specificity (73.9%, 75.8%; *P *= 0.39). The study shows that customizing MRI protocols can shorten exam time without sacrificing diagnostic accuracy, offering a potential approach for optimizing future MRI screening as it expands accessibilities.^81^

**Fig. 5:**
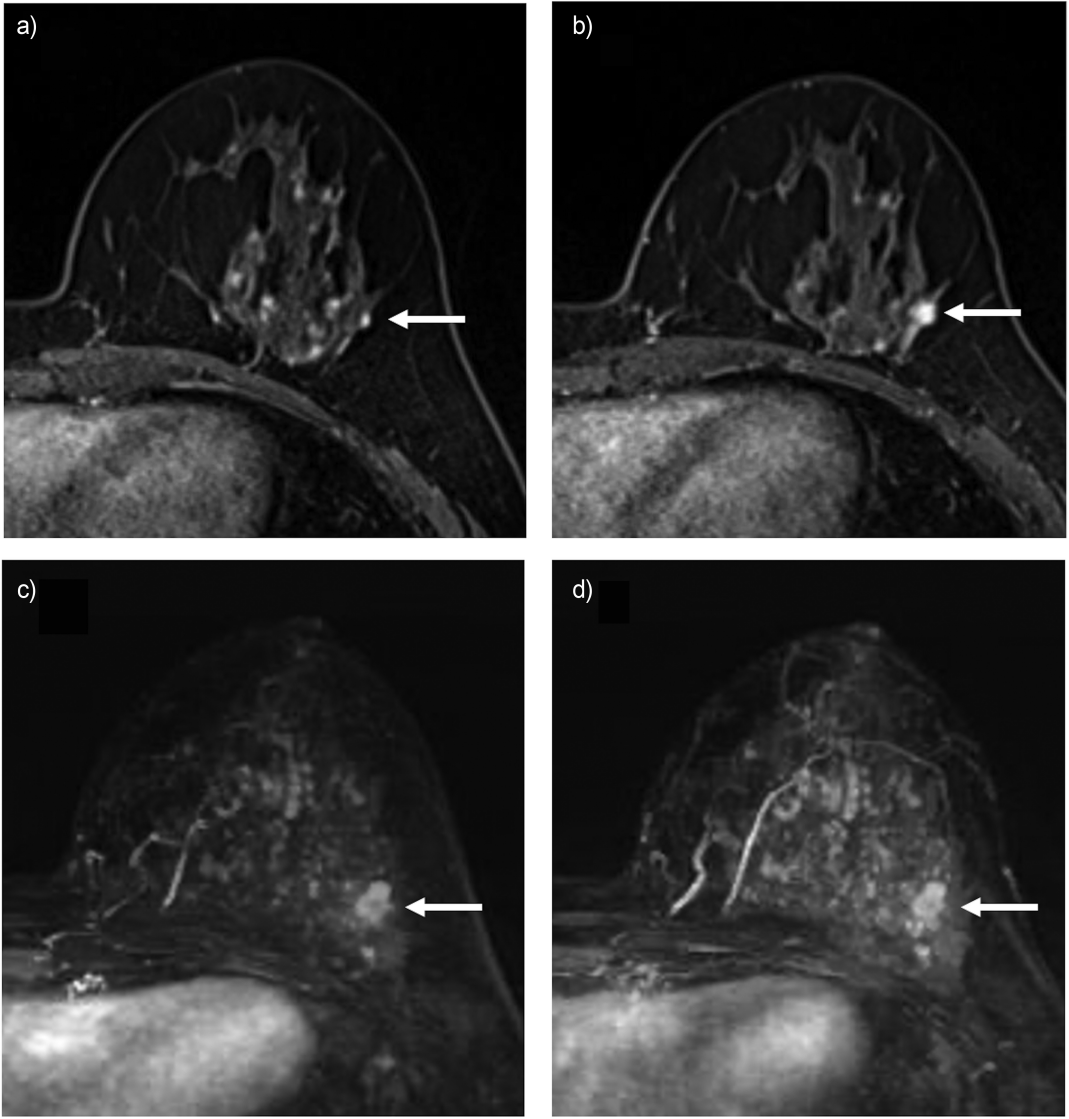
Woman with a pathogenic variant in *BRCA1* and a right breast-conserving surgery at her 30s. She started imaging screening using yearly MRIs after her operation. (**a**) and (**b**) are the 2nd phase of DCE-MRI. (**a**) A mammogram was assessed as “normal” 1 year before the lesion was suspected. (**b**) Due to the enlargement of an 8 mm enhancing lesion in the left outer lower quadrant region of the breast, the lesion was diagnosed as BI-RADS category 4A. The US followed up on the lesion, and 7 months later, a suspicious lesion was detected in the US and biopsied. The biopsy showed a luminal B type invasive ductal carcinoma. This was diagnosed 10 years after she got her first cancer. Then, preoperative MRI was performed. (**c**) is the 10th phase and (**d**) is the 20th phase of the UF DCE-MRI. The lesion is visible in the early phase of UF DCE-MRI with less BPE. The white arrows in each image indicate the lesion. BI-RADS, Breast Imaging Reporting and Data system; BPE, background parenchymal enhancement; DCE-MRI, dynamic contrast-enhanced MRI; US, ultrasound, UF, ultrafast.

Figure 5 shows a representative case of a woman who was suspected of having cancer on annual MRI screening for high-risk women. The contrast-enhancing lesion seen as BPE was noted to have increased in size in the following image and was described as BI-RADS Category 4, to be biopsied. The lesion was diagnosed as invasive ductal cancer. UF protocol was performed, and the lesion showed a visible enhancement easily differentiated from BPE.

### Potential alternatives to other modalities

DCE-MRI is an efficient modality for annual screening of high-risk women; however, the problem is its limited availability, high costs, and restricted patient use for those who have an allergy to contrast agents or contraindications to undergo MRI, such as non-MRI-compliant implants and claustrophobia. When implementing high-risk MRI screening for worldwide use, one may also consider other screening possibilities, such as accessible and effective mammography without MRI. Recently, newly developed mammography techniques have been highlighted, especially for women with dense breasts, to overcome the weakness of mammography screening: digital breast tomosynthesis (DBT), contrast-enhanced digital mammography (CE-DM), and contrast-enhanced digital breast tomosynthesis (CE-DBT).

DBT consists of multi-layer images focusing on specific depths of the breast tissue, diminishing the effect of overlapping dense breast tissue using a 3D projection.^82^^,^^83^ This technology is now approved and implemented for routine clinical use and can potentially replace traditional mammography screening. A systematic review of 26 articles on the efficacy of DBT in women with dense breasts and additional risk factors found that DBT + DM may be more accurate compared to DM (82.8%–92.5%, 56.8%–81.3%).^84^ A cohort study at imaging facilities affiliated with the Breast Cancer Surveillance Consortium (BCSC) evaluated evidence on DBT and DM screening outcomes among 208945 women with a family history. The study demonstrated that DBT screening reduced recall rates and improved specificity compared to DM screening, especially in women with a first-degree relative.^85^

CE-DM is a mammography-based technique that allows neo-angiogenesis information to be acquired using an iodine contrast agent. This technique utilizes dual-energy technology for low and high-energy acquisition with subtraction images to visualize the enhancing areas.^86^ The strength of CE-DM is that it can obtain both morphological and vascular information. CE-DM may have better sensitivity and specificity compared with DM alone or mammography + ultrasound, especially in patients with dense breasts. A meta-analysis showed that CE-DM and DCE-MRI possess comparable diagnostic performance; DCE-MRI surpasses CE-DM in the most clinically significant metrics: sensitivity, negative predictive value, and negative likelihood ratio.^87^ Also recently, results from the Breast screening—Risk Adaptive Imaging for Density (BRAID) randomized controlled trial that directly compared supplemental screening methods for intermediate-risk women with dense breasts with negative mammogram, including abbreviated MRI, automated whole breast ultrasound (ABUS), and CE-DM, have been published. Abbreviated MRI and CE-DM detected 3 times more invasive cancers than ABUS, with the tumors half the size. Detection rates showed no significant difference between CE-DM and abbreviated MRI (*P* = 0.62).^88^ Furthermore, a randomized controlled trial (DENSE2 trial) is ongoing to compare 2 interventional arms, CE-DM and abbreviated MRI, each combined with mammography.^89^

CE-DBT provides 3D mammographic images with contrast enhancement, potentially improving lesion characterization and localization. Research on this topic remains limited, with a study focusing on a modality characteristic.^90^ Although it provides slightly lower contrast enhancement than CE-DM, it provides better lesion margins. One study assessed women with BI-RADS 4 or 5 lesions to compare the diagnostic accuracy of DCE-MRI, CE-DBT, CE-DM, DBT, and DM, reporting ROC values of 0.897, 0.892, 0.878, 0.784, and 0.740, respectively.^91^ They concluded that CE-DBT and CE-DM may serve as alternative modalities to MRI for follow-up in women with abnormal mammography findings. However, iodine-based contrast agents (used in CE-DM) pose a higher risk than gadolinium-based contrast agents used in DCE-MRI, so DCE-MRI should be preferred over CE-DM in patients with a history of allergic reactions.

Although the evidence of the diagnostic ability of these modalities is insufficient, it may be useful as an alternative to DCE-MRI for screening high-risk women when DCE-MRI is not available or not suitable, especially in women with dense breasts.

## The Long-term Outcomes of High-risk Breast Cancer Screening

Assessing the effects of screening, including breast cancer mortality as an endpoint, requires a long period of follow-up and a large sample size. The guidelines are gradually being developed and implemented in many institutions. Long-term prognosis related to MRI screening has become available. In this section, we describe the literature in the order of its published year. Table 1 shows studies that evaluated the long-term outcomes of MRI screening for high-risk women.

In 2013, a Norwegian study reported the 10 year survival as 69% [95% CI, 48%–83%] for women diagnosed with breast cancer in the annual MRI surveillance program for *BRCA1* carriers.^92^ In 2015, studies from the Netherlands, UK, and Canada analyzed 1275 *BRCA1/2* carriers with 124 cancers, showing MRI plus mammography combined group reduced breast cancer mortality by 50%–62%, compared to 42%–47% with mammography alone.^93^ In the same year, Saadatmand et al. compared metastasis-free survival in *BRCA* carriers and women with familial risk (15%–50% lifetime risk) screened by MRI and mammography versus matched controls (average-risk women) screened only by mammography.^94^ Metastases occurred in 9% of cases in the MRI group versus 23% in controls (*P* = 0.009), with 10 year metastasis-free survival rates of 90% and 77%, respectively (*P* = 0.008).

In 2020, Bae et al. retrospectively compared outcomes in women screened with MRI plus mammography (n = 1534) vs. mammography alone (n = 1468) over a median of 10.9 years.^95^ The combined group had a higher cancer detection rate (1.4% vs. 0.5%, *P* < 0.001) and better OS rate (*P* < 0.002), but no significant difference in disease-free survival rate (*P* = 0.32).

In 2021, Evans et al. reported on 33 years of enhanced screening (annual mammography from 1987, MR from 1997) in 14311 women with an increased lifetime risk of breast cancer (≥17% lifetime risk), detecting 649 cancers—394 through screening, with 68.5% at early stages. 23.5% were identified as interval cancers, 7 of which were identified by RRM.^96^ Interval cancers showed lower 10-year survival than screen-detected ones (80.2% vs. 91.9%, *P* < 0.001). These cancers were expected to exhibit characteristics of the *BRCA1*-variant, grade 3, ER negative and HER2 negative more frequently than the high-risk non-*BRCA* group (*P* < 0.0001).^97^ According to the study, most deaths from TNBC occurred within the first 5 years. However, a study from Italy suggests that the reported survival gap between TNBC and non-TNBC can be reduced by MRI screening.^98^ The study analyzed asymptomatic high-risk women diagnosed with invasive breast cancer during MRI screening over 9.7 years, comparing 14 TNBC and 30 non-TNBC cases. There were no significant differences in the 5-year OS rates (86% vs. 93%, *P* = 0.95). In contrast, a study conducted in Toronto found that the 5-year survival rate for TNBC mammography-only screening was approximately 70%.^99^ The authors speculated that improvements in survival may stem from earlier cancer detection, *BRCA1* cancer sensitivity to chemotherapy, effective treatments, and potential bias from proactive high-risk participants in screening and treatment.

In 2024, Lubinski et al. also evaluated groups of MRI plus mammography and only mammography surveillance using an extensive prospective survey that included 2488 women, combining data from 59 centers in 11 countries.^100^ Women undergoing MRI surveillance showed a significant reduction of mortality with an age-adjusted HR of 0.20 (95% CI, 0.1*–*0.43; *P *< 0.001) in *BRCA1* carriers compared to those without MRI surveillance. However, *BRCA2* carriers did not show a significant reduction, with an HR of 0.87 (95% CI, 0.10*–*17.2; *P *= 0.93). The estimated cumulative risk of breast cancer mortality by the age of 75 was 20.5% for individuals who did not undergo MRI surveillance, compared to 5.5% for those who did (*P* < 0.001).

Finally, in contrast to the report by Lubinski et al., the Evans result showed no significant difference in the breast cancer-specific survival rate of *BRCA* carriers and non-carriers. Still, the curve of the *BRCA2* carrier crossed that of the *BRCA1* carrier after 10 years (20 years survival of *BRCA1* 91.5%, *BRCA2* 85.1%, and non-*BRCA* 84.7%). The lower breast cancer-specific survival rate for *BRCA2* compared with *BRCA1*, which is considered to have a poorer prognosis, may provide a glimpse into the proper long-term prognosis of *BRCA2* but needs further investigation. Compared to the study from 2013,^92^ 10-year breast cancer-specific survival rates have improved in the studies in 2021 and 2024,^96^^,^^100^ possibly due to advances in imaging and treatment technology over the past 10 years (Table 1).

## Impact of Screening Adherence in High-risk Populations

The mortality rate from breast cancer has been increasing globally over the past 25 years, especially in developing and low-income regions, with approximately 7 additional cases per million people every 5 years worldwide.^101^ On the other hand, breast cancer mortality rates are decreasing in Western Europe, and the main reason for this is the increased early cancer detection due to screening and the availability of more effective treatment. The upward trend in the mortality rate from breast cancer in Japan has recently begun to slow down.^102^ A prerequisite for an effect on mortality is that women regularly participate in screening. The rate of participation in screening is affected by women’s awareness and understanding, and availability to access the screening, with long-term, continuous participation being a bottleneck.

Lower sociodemographic residential status is associated with both lower participation in screening and worse breast cancer prognosis. Looking at screening in average-risk women, a 2021 meta-analysis of 66 studies showed that those with economic and social advantages had higher mammography participation rates.^103^ Additionally, poor health habits and lack of a history of breast cancer were also linked to non-participation.^104^ Epidemiological studies show that, after adjusting for age, year, and stage at diagnosis, women with high education had a 35% lower 5-year breast cancer mortality risk (HR =  0.65; 95% CI, 0.53*–*0.80) compared to that of low education,^105^ and those from higher-income households had a 19% lower 10-year risk (HR =  0.81; 95% CI, 0.67*–*0.97) compared to that of lower income households.^106^ These disparities likely reflect both short-term health differences and long-term inequalities in cancer care. In addition, a recent study found that women with screen-detected breast cancer are more likely to experience delayed diagnosis if they live in rural areas compared to urban areas.^107^ To improve equity in healthcare, the impact of sociodemographic and residential background in relation to screening participation rates needs to be investigated.

Within the scope of our study, no previous studies have tracked the sociodemographic background of women who participated in high-risk screening. Several studies have examined factors that influence the decision of women to undergo surveillance or opt for RRM. Women undergoing RRM are more likely to have children; their first-degree family member has a history of breast cancer; have higher education; and have undergone RRSO.^108^^–^^110^ Finally, research about women’s perceptions of MRI surveillance suggested that participants live in a cycle of coping with emotions and structuring their lives based on surveillance.^111^ Supporting decision-making and person-centered care could be essential to increasing sustainable surveillance participation in the future.

## High-risk Screening for the Japanese Population

The 2018 Japanese Breast Cancer Society guideline recommended adding contrast-enhanced breast MRI to annual mammography for *BRCA1/2* mutation carriers.^112^ In April 2020, Japan introduced insurance coverage for genetic counseling and testing only for women with a history of breast cancer who met specific criteria: diagnosis before age 45, TNBC before age 70, multiple breast cancers, a third-degree relative with breast, ovarian, or pancreatic cancer, a close relative with a *BRCA1/2* mutation or male breast cancer, or a prior diagnosis of ovarian, fallopian tube, or peritoneal cancer. Women with a history of breast cancer who are found to carry a *BRCA1* or *BRCA2* mutation are eligible for insurance coverage for RRM and RRSO. It is an important step forward for women at high risk in Japan. Some institutions had already begun providing support before the insurance was introduced, but participation increased after its introduction. However, few studies in Japan have examined screening strategies for high-risk women.^32^^,^^113^^,^^114^

According to the Organization for Economic Co-operation and Development (OECD) statistics, Japan has the most MRI scanners per million people (57.4), followed by the US (38.0), Korea (35.5), Germany (35.3), and other developed countries; however, developing countries have less than 0.3.^115^ Furthermore, under comparable personal and family histories of breast and/or ovarian cancer, the Japanese population demonstrates a higher prevalence of *BRCA1/2* pathogenic variants than the general non-Ashkenazi population (odds ratio = 1.87).^116^ The higher prevalence may be explained by the selection bias for using populations recruited from genetic counseling, or by the presence of common *BRCA2* pathogenic variants among Japanese.^117^ Japan is uniquely positioned to potentially contribute valuable research in this field with its well-established MRI infrastructure and higher penetrance of *BRCA1/2* pathogenic variants.

## Conclusion

With increased awareness and improved genetic testing, more individuals carrying the *BRCA1/2* variants or other breast cancer-related pathogenic variants are being identified. This has led to a growing demand for preventive procedures like RRM, oophorectomy, and MRI screening. MRI has significantly improved early detection in high-risk women, potentially reducing cancer progression and improving long-term outcomes. There is a call for personalized, cost-effective screening methods that consider individual risk factors and address socioeconomic disparities to ensure sustainable screening strategies.

## Figures and Tables

**Table 1:** Long-term outcomes of high-risk screening

Year	Country	Ref.	Design	Time Frame	Inclusion risks	Study Population			Study Results					
						Total women	Study or Control Arms		BC (IC)	BC death	BC-specific survival rate (%)		BC Metastasis	Metastasis-free survival rate (%)
							Type	Total			5y	10y		
2013	Norway	92	P	2001–2011	*BRCA1*	802	MRI + MG		68 (63)	10	75.0	69.0		
2015	Netherlands	94	P	1999–2007	*BRCA1/2,* familiy risk	4616	MRI + MG	2308	93	7			8	90
							MG only^*^	2308	93	19			21	77
2020	USA	95	R	2001-2004	*BRCA1/2*	3002	MRI + MG	1534	38 (40)	0			6	
							MG only	1468	22 (23)	5			5	
2021	UK	96	P	1987–2020	>17% LTR	14311	MRI + MG		394 (322)	54	94.1	91.0		
2024	USA + 10	100	P	1995–2022	*BRCA1/2*	2488	MRI + MG	1756	241 (205)	14		93.8		
							MG only	732	103 (79)	21		86.7		

BC, breast cancer; IC, invasive cancer; LTR, lifetime risk; MG, mammography; P, a prospective study; Ref., reference number; R, a retrospective study.

* Control arms: unscreened if under 50 years and screened with biennial mammography if over 50 years.
